# Integrated High Resolution Digital Color Light Sensor in 130 nm CMOS Technology

**DOI:** 10.3390/s150717786

**Published:** 2015-07-22

**Authors:** Drago Strle, Uroš Nahtigal, Graciele Batistell, Vincent Chi Zhang, Erwin Ofner, Andrea Fant, Johannes Sturm

**Affiliations:** 1 University of Ljubljana, Electrical Engineering Department, Tržaška 25, Ljubljana 1000, Slovenia; E-Mail: uroš.nahtigal@fe.uni-lj.si; 2 Carinthia University of Applied Sciences, Europastrasse 4, Villach 9585, Austria; E-Mails: g.batistell@fh-kaernten.at (G.B.); j.sturm@fh-kaernten.at (J.S.); E.Ofner@fh-kaernten.at (E.O.); 3 Infineon Technologies Austria AG, Siemensstrae 2, Villach 9500, Austria; E-Mails: VincentChi.Zhang@infineon.com (V.C.Z.); andrea.fant@infineon.com (A.F.)

**Keywords:** color light detection, high-resolution photo current measurements, measurements of light in CIE XYZ color luminosity space, CMOS-integrated color light sensor system, RGB color sensor, color signal processing

## Abstract

This article presents a color light detection system integrated in 130 nm CMOS technology. The sensors and corresponding electronics detect light in a CIE XYZ color luminosity space using on-chip integrated sensors without any additional process steps, high-resolution analog-to-digital converter, and dedicated DSP algorithm. The sensor consists of a set of laterally arranged integrated photodiodes that are partly covered by metal, where color separation between the photodiodes is achieved by lateral carrier diffusion together with wavelength-dependent absorption. A high resolution, hybrid, ∑∆ ADC converts each photo diode’s current into a 22-bit digital result, canceling the dark current of the photo diodes. The digital results are further processed by the DSP, which calculates normalized XYZ or RGB color and intensity parameters using linear transformations of the three photo diode responses by multiplication of the data with a transformation matrix, where the coefficients are extracted by training in combination with a pseudo-inverse operation and the least-mean square approximation. The sensor system detects the color light parameters with 22-bit accuracy, consumes less than 60 μA on average at 10 readings per second, and occupies approx. 0.8 mm^2^ of silicon area (including three photodiodes and the analog part of the ADC). The DSP is currently implemented on FPGA.

## 1. Introduction

Colorimetric light measurements are typically based on three independent optical sensors with a defined spectral responsivity behavior that matches the CIE color matching functions (CMF) [1]. Different color matching functions like R, G, B or X, Y, Z were defined by experiments and empirical observation according to the human eye’s color perception. The Y component of the X, Y, Z color model matches the V(λ) curve, which represents the luminance of the color. The color matching functions can be seen as reference curves which should be matched to the color sensor spectral sensitivity characteristics in order to accurately represent a light color.

Standard silicon photodetectors in an integrated circuit technology are usually realized as reverse biased pn-junctions, and show a much wider spectral responsivity compared to the CMF. Therefore, the spectral response of silicon photodiode sensors needs to be optimized. A classical method is the implementation of expensive narrow-band color filters (Bayer Color Filter Array-CFA) on top of wide-band silicon photodetectors [2]. Even though these color filters are widely used, they have also drawbacks since the filters significantly decrease the overall sensor sensitivity by absorbing most of the incident light. Furthermore, these sensors require a complex filter assembly on top of the sensor surface, which significantly increases the production costs. Alternatively, to external filter-based sensors, also filter-less sensor concepts are used, mainly based on the wavelength-dependent light absorption properties of silicon [3,4,5,6,7,8,9]. A stack of optimized vertically arranged pn-junctions can be used, where each one absorbs the generated carriers related to different light wavelengths. Therefore, the sensor responses can be modified, in order to be sensitive to different wavelengths of the visible light range. Since absorption losses are avoided with this sensor structure, a better quantum efficiency is achieved compared to the sensors with color filters. The spectral responsivity of stacked photodiodes is directly dependent on the technology parameters like doping concentrations and profiles. Even though modern CMOS technologies include a triple-well option—which provides three laterally stacked pn-junctions in different depths that enable color recognition—there is only a limited room for optimization of the spectral response without expensive technology modifications. Also, for older and therefore cheaper CMOS technologies, a triple-well option is not always available. A sensor in a low-cost technology without triple-well and process modification, but only with optimization of the spectral sensor response by layout modifications is proposed in this paper. Another method for tuning the sensors response, based on the generation of high electric fields in a widely depleted epitaxial layer of a CMOS technology, is reported in [10,11]. The disadvantage of this sensor is the required low-doped epitaxial layer, which is not part of a standard CMOS process flow.

The main target of the proposed photosensor design is monolithic integration in a standard, low-cost CMOS technology, without any process modifications, together with analog and digital signal processing electronics, providing three spectrally independent output signals, for example, RGB. In order to avoid filters and expensive multi-diode stacks, the proposed color detection methods are based on wavelength-dependent light absorption, together with lateral carrier diffusion effects and complex analog and digital signal processing. The realization and optimization of the sensor is done by layout modifications of the standard CMOS process only. The proposed color detector includes three independent sensors with different spectral sensitivities. Even though the spectral sensor responsivities do not perfectly match the R,G,B or X,Y,Z color matching functions, the sensor output signals can be optimized by digital signal processing after analog-to-digital conversion. Matrix operations can be performed to transform the sensor output signals to the standardized R,G,B or X,Y,Z tri-stimulus values. An optimized transformation matrix has to be extracted based on training strategies and pseudo-inverse matrix operations to minimize the least-mean-square error of the signal transformation.

Each photocurrent must be converted to a digital word in as short time as possible, keeping the average power consumption as low as possible. If enough time is available and if the application allows, the time multiplexing is used to convert three currents. For modest resolution requirements of up to 16 bits, and if a long conversion time is allowed, an integrating ADC can be used [12]. For higher resolution and a shorter conversion time, a ΣΔ [13] or incremental [14] converter may be used. In our case, the evaluation of the color signal-processing algorithm reveals that the resolution of digital data representing photocurrents must be higher than 20 bits for accurate calculation of the spectral components of the light.

The paper is organised as follows: Section 2 describes the principles of the integrated light-to-digital converter. In Section 3, the theoretical background of the proposed sensor structure is presented together with device and process simulations. Section 4 presents the architecture, design, modeling, and simulations together with measurement results of a complete photo-current-to-digital converter. Section 5 describes the fundamentals of the color-processing algorithm and its DSP implementation on the FPGA. Section 6 presents some experiments and measured results, while Section 7 concludes the article.

## 2. Principles of Operation

**Figure 1:**
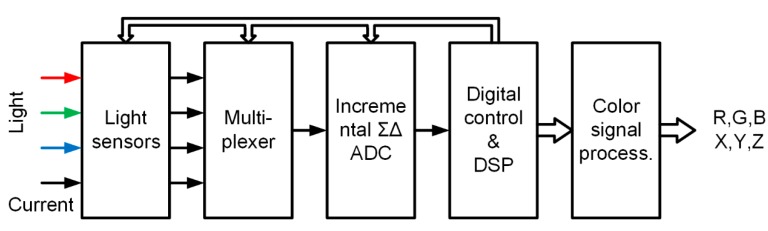
Color measurement process.

The simplified block diagram of a complete light-to-digital detection system is presented in Figure 1. Light is sensed by three pairs of photo diodes with different responsivities (Light Sensor in Figure 1) [15]. One diode in a pair is dark (covered with metal), and one is active (open to the light). The pairs of photocurrents are time multiplexed. Each photocurrent pair is converted into a 22-bit digital word, using a hybrid ΣΔ analog to digital converter (ADC); each conversion takes 2 ms. The function of the DSP includes the control of the analog-front-end (AFE), multiplexing and attenuation of the out-of-band quantization noise, and it also reduces the sampling rate. In addition, it removes the remaining offset voltage, 1/f noise and “dark currents” from the photo diodes and executes the color signal processing algorithm that generates color elements, according to the CIE standard.

## 3. Color Sensor Design and Measurements

### 3.1. Proposed Photo Sensor Principles

Different methods for the modification of spectral responses of integrated photodiodes in a standard CMOS technology are possible. A standard pn-junction photodiode shows a very wide spectral response, with a broad sensitivity of the visible light range, which makes it not well-suited for color discrimination. Lateral carrier diffusion effects can be used to create a sensor, which is mainly sensitive to longer wavelengths (red light). The retrograde p-well of a CMOS process can help to shield substrate carriers, which enables photodiodes with a responsivity maximum at low wavelengths, making them sensitive to blue light. With these methods, it is possible to create photodiodes with at least three different independent spectral sensitivities, which is necessary for light color recognition.

#### 3.1.1. “Red” Spectral Response Sensor

**Figure 2:**
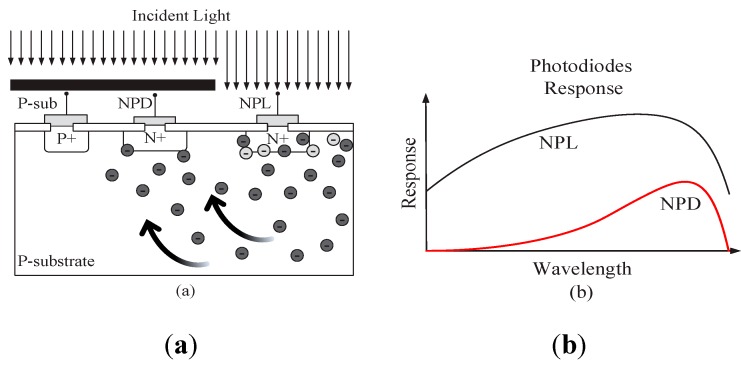
(**a**) Illustration of carrier generation and lateral carrier diffusion for short (light grey carriers) and long light wavelengths (dark grey carriers); (**b**) Effect of lateral carrier diffusion on photodiodes responsivities.

Figure 2a shows a silicon photodetector structure with two pn-junctions (NPD and NPL) implemented as highly doped N+ regions in a p-doped silicon substrate without an epi-layer [15]. Metal plate shields the NPD photodiode from incidental light, while the other (NPL) is directly exposed to the light. When the light is absorbed, minority carriers are created within the silicon substrate. Due to diffusion and drift effects, the minority carriers move within the silicon substrate until they recombine or are collected by a photodiode pn-junction, so the photodiode reverse current is increased. The carrier generation rate decreases exponentially with the depth of the silicon bulk, depending on the light wavelength. Low light wavelengths of about 400 nm to 500 nm (“blue”) create carriers near the silicon surface (light grey) as indicated in Figure 2a. Visible light with a longer wavelength, above 600 nm, (“red”) has a higher penetration depth, and with a high carrier generation deep in the silicon (dark grey). Depending on the penetration depth, a certain number of carriers are reaching the pn-junctions of the covered photodiode NPD, as well as the uncovered photodiode NPL due to lateral carrier diffusion. This results in different spectral responses for the two photodiodes as shown in Figure 2b. The NPL is sensitive to a wide spectral range, while the NPD has a maximum sensitivity at long wavelengths (“red”).

#### 3.1.2. “Blue” Spectral Response Sensor

**Figure 3:**
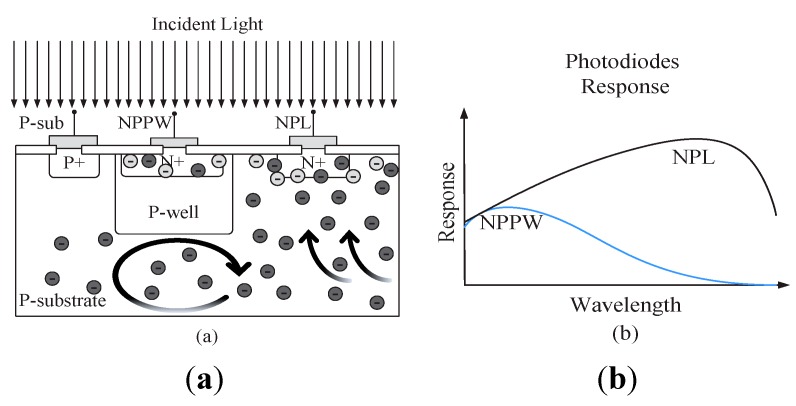
(**a**) A retrograde p-well can be used to shield substrate carriers; (**b**) The responsivity of the shielded pn-junction NPPW is sensitive to short (blue) light wavelengths.

Two shallow pn-junctions of a N+ implant in a P-well (NPPW) and a N+ implant directly in P-substrate (NPL) are shown in Figure 3a. When broadband (“white”) light falls on the silicon surface, carriers are generated near the surface as well as deep in the substrate. The carriers are able to diffuse from the substrate directly to the photodiode NPL and create a photocurrent with a broad spectral responsivity, as shown in Figure 3b. For the second photodiode NPPW, the carriers from the substrate are shielded by the special doping profile of the retrograde P-well layer. Since carriers created by long light wavelengths are shielded, the spectral response of the photodiode NPPW shows a responsivity maximum at low wavelengths (“blue”). Even though both NPL responses in Figure 2b and Figure 3b show a wide spectral sensitivity, they are slightly different at low and high wavelengths respectively, since the integrated sum of both diodes in Figure 2b and Figure 3b is approximately the same.

### 3.2. Proposed Color Sensor Implementation

**Figure 4:**
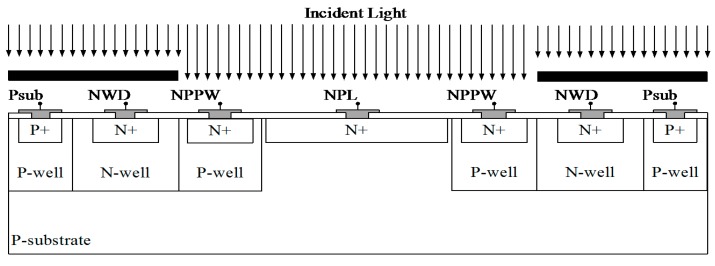
Cross-section of the proposed color sensor structure with three-sensor output signals NPL, NWD, and NPPW with different spectral responsivities.

All three photodiodes from the previous subsection are combined in a color sensor structure shown in Figure 4 [16,17]. The whole sensor is based on standard 130 nm CMOS technology without using any process modification. It is implemented in a p-substrate connected to a ground through p+/p-well implants (Psub). Two pn-junctions (NWD) are covered by metal to shield the diodes from direct light exposure leading to a “red” spectral response. Compared to Figure 2a, a deeper n-well junction is used instead of a shallow highly doped N+ region to improve the diffusion carrier collection efficiency. The two NWD diodes are implemented as stripes with 3.5 μm width and 250 μm lengths. A “wide” spectral response is obtained by the photodiode NPL that has an uncovered pn-junction between the n+ implant and the p-substrate with 14 μm width and 250 μm lengths. The response to short wavelengths (“blue”) is obtained by two pn-junctions created between an n+ implant and a p-well (NPPW) with a size of 12 μm × 250 μm.

#### 3.2.1. Color Sensor Simulations

The photodiode device and process simulations were performed with a Synopsys TCAD Environment. The influence of the photodiodes dimensions, and arrangement as well as the metal coverage was investigated and the design was optimized by simulation. A Synopsys TSUPREM-4 was used to perform a 2-dimensional simulation of the 130 nm CMOS production process. The optical and electrical simulations were performed with a Sentaurus Device using the drift-diffusion transport model. The optical generation rate is calculated using Raytracer as an optical solver, which is optimized for the high simulation speed. The Raytracer was implemented based on linear polarization and geometric optics, where the light propagation is described as rays. The Raytracer uses a wavelength-dependent complex refractive index model. The simulation tools use the Shockley-Read-Hall (SRH) model to calculate the recombination rate. In the SRH, the doping dependency of the carrier lifetime was considered, while the temperature and electric field dependency were neglected. The connection between the electrical and optical simulations is provided by including the carrier generation rate and recombination rate in the continuity equation. Furthermore, the carrier mobility is defined as doping-dependent. Due to the high simulation time of 3-dimensional models, most simulations are based on 2-dimensional solvers. The proposed detector consists of five color sensor stripes connected in parallel, as shown in Figure 15. Therefore, a 2-dimensional simulation of a single sensor stripe gives only slightly different results compared to a 3-dimensional simulation. As a good approximation, the responsivity difference can be corrected by a constant factor. After finding the initial solution, the quasi-stationary equations are solved by ramping the wavelength of an ideal monochromatic light source from 400 to 1000 nm. The sensitivity of each photodiode is analyzed by its responsivity. The responsivity is defined by the diode photocurrent divided by the applied light power at a specific wavelength. All simulations were done with ideal electrical contacts biased at 0 V dc-voltage. Parasitic charges within the oxide and the silicon/oxide interface might cause leakage and inversion problems, so they are considered during the device simulations. To avoid edge effects in the simulations, the silicon substrate depth was increased to 100 µm. 

**Figure 5:**
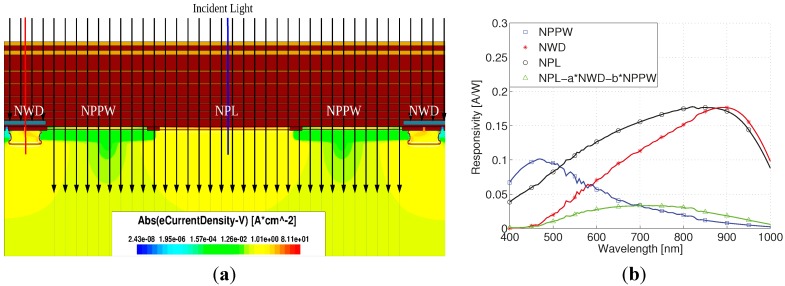
(**a**) Simulated electron current density of the color sensor structure; (**b**) Simulation results of the responsivity of uncovered NPL and NWD photodiodes without the influence of the oxide/dielectric stack.

The color detector cross section including simulated electron current density for a light wavelength of 900 nm is presented in Figure 5a. Although the light only falls on the center, there is also a significant electron current density at the covered n-well due to lateral carrier diffusion, as previously discussed. The detector responsivity simulations are presented in Figure 5b. The NWD is sensitive to long wavelengths, the NPPW is sensitive to short wavelengths, and the NPL has a wide spectral response. A responsivity sensitive to middle wavelengths, can be obtained by a scaled subtraction of NWD and NPPW from NPL with scaling factors a = 0.6 and b = 0.55. The simulation results in Figure 5b neglect the diffraction and interference effects caused by the oxide/nitride stack of the metallization processes shown on top of the silicon substrate in the detector cross section in Figure 4.

Even though the presented structure contains just simple lateral photodiodes, the responsivities are comparable to the stacked photodiodes presented in [18,19,20], and therefore the three sensor outputs can be directly used for color measurement

## 4. High Resolution Light to Digital Conversion

### 4.1. Photocurrent to Digital Converter

**Figure 6:**
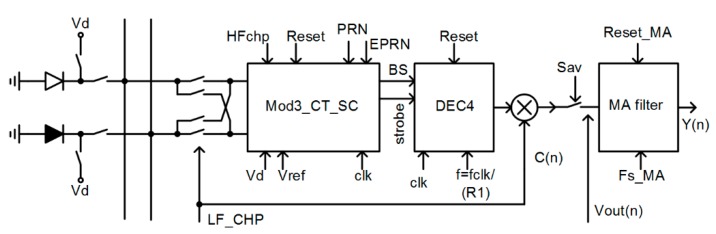
Simplified block diagram of the light-to-digital conversion.

High accuracy color signal processing requires high-resolution light-to-digital conversion, as stated in Section 5. In addition, the dynamic range of the light is extremely high, so a photocurrent can be in a range from pA, at a small light intensity, up to several μA and more in full light conditions. This means that a dynamic range (DR) of over 120 dB is required, and so high resolution ADC is needed. Integrating ADC [21] can be used for modest requirements regarding dynamic range, a resolution (up to 16 bits), and conversion rate of up to 100 ms. For extreme requirements regarding DR, accuracy and speed, the possible architecture is current mode incremental ΣΔ ADC, which can handle a huge dynamic range, reduce the influence of the dark current of the photodiodes, and remove the offset voltage and the 1/f noise of the analog integrators in a relatively short time (2 ms). The architecture is presented in Figure 6. A pair of photo-diodes is shown on the left, although in a real circuit three pairs of multiplexed photo-diodes (NPL, NWD, and NPPW) are used; they are sensitive to different light spectra, as explained in Section 3. The active photo diode (white on the left part of Figure 6) converts light into a photocurrent. The dark current of each active photo-diode is compensated with a current from the “dark” photo-diode (black on Figure 6). The diodes are very close to each other on the same silicon substrate (see Figure 4). Their layout and the technology steps are equal except for the metal cover on top of the dark photo-diode. 

The temperatures of both photo-diodes are the same if the layout of the whole chip is appropriate, so they behave electrically equal and their dark-currents are approx. equal. The effective current flowing into the current-to-digital converter is the difference of both currents $I_{photo}=I_{A}-I_{D}$ ($I_{A}$ is a photo current of the active photodiode, $I_{D}$ is the dark current), which is converted into a 22-bit digital result in 2 ms. The whole circuit is composed of a 3rd order, hybrid, incremental ΣΔ modulator (Mod3_CT_SC on Figure 6) running with an approx. 2 MHz oversampling rate followed by a 4th order Sinc decimation filter [22] (DEC4 on Figure 6) with an oversampling ratio of R=512, and with the transfer function given in Equation (1):

(1) $$H(z)={\left[\sum_{n=0}^{R}z^{-n}\right]}^{4}={\left[\left(\frac{1-z^{-R}}{1-z^{-1}}\right)\right]}^{4}$$

To remove the offset voltage and flicker noise of the first integrator, a high frequency chopping (HFchp signal) is used in the opamp of the first integrator. The mixing translates the offset voltage and 1/f noise of the opamp, around ½ of the over-sampling frequency, which must be above 1/f noise corner frequency of the CMOS process. In addition, photocurrents are “chopped” using a multiplexer driven by the signal LF_CHP, presented on the left part of Figure 6. Chopping translates both photocurrents to the spectral component around frequency *f_LF_chp_* ≅2 kHz. A 24-bit decimator output signal is digitally mixed with signal C(n)=LF_CHP; it translates the main spectral line to the DC, while the remaining offset voltage of the complete modulator is up-converted to the spectral line at frequency *f_LF_chp_*; the MA filter removes this spectral component. In this way, a special kind of nested chopper technique is implemented, which offers the best results regarding the offset and the 1/f noise. The residuals of the LF chopping spectra are removed in a first order moving average filter (MA_filter in Figure 6 that calculates the average of the two consecutive results of the decimation filter output according to Equation (2), where $y_{n}=k\cdot I_{photo}+V_{off}$ and $y_{n-1}=-k\cdot I_{photo}+V_{off}$:

(2) $$y_{m}=\left(y_{n}-y_{n-1}\right)/2$$

The MA filter also reduces the word-length at the output to 22 bits. Every conversion starts with a reset of all integrators in the CT and SC part of the modulator, and all the registers in the decimation filter, and continues with a conversion for 4 × 512 periods of the oversampling clock; the result $y_{n-1}$ is stored in one register of the MA filter. In the second phase of the conversion, the sign of the input current changes; all integrators are reset, the inverted photo current is converted as before, and the results are stored in $y_{n}$ of the MA filter. At the end, the MA filter calculates the result using Equation (2). $Y_{m}\left(z\right)$ in Equation (3) represents the digital results in the z-domain, where *k* is a constant dependent on the reference voltage and the current/capacitor ratios of the modulator, while the sampling period is equal to the period of the LF_CHP signal: $f_{LF\_chp}=f_{ovs}/\left(R\cdot 8\right),$ where $f_{ovs}$ is the frequency of the oversampling clock, and R is the oversampling ratio. The factor 8 in the denominator comes from the fact that decimation filter needs 4 × R periods to calculate stable results after the reset; two such periods are needed for one result. The dark current, offset voltage, and 1/f noise voltage are thus completely removed from the digital results of Equations (2) and (3).

(3) $$Y_{m}\left(z\right)=k\cdot I_{photo}\left(z\right)\cdot \left(1+z^{-1}\right)$$

**Figure 7:**
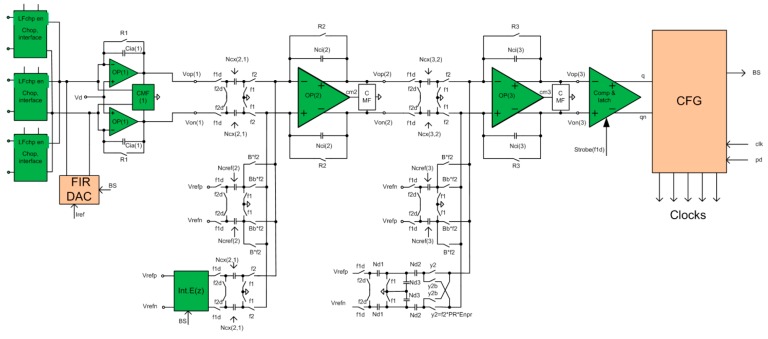
A simplified circuit diagram of a hybrid, 3rd order ΣΔ modulator with multi-bit FIR-DAC and a corresponding compensation circuit IntEz. It also includes a multiplexer where three pairs of photo-diodes are connected in addition to an input for testing purposes.

**Figure 8:**
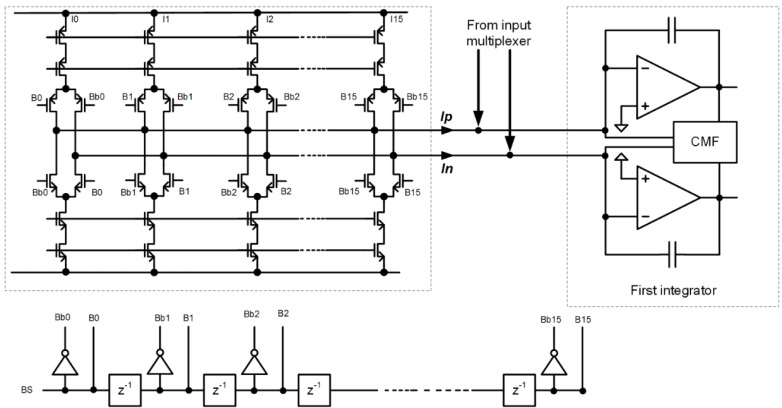
FIR-DAC filter implementation. The weights are implemented by current sources with different ratios. The reference current is possible to adjust to compensate inaccuracy due to matching.

To satisfy the high dynamic range, linearity, and resolution requirements with a limited possibility to feed-forward the photo current, a third order modulator with a single-bit internal quantizer was selected. The coefficients of the discrete time model were synthesized using the delsig toolbox from Mathworks [23]. Since input information to the converter is photo-current, a hybrid continuous-time (CT)-discrete-time (DT) modulator was selected, where the first integrator is CT, while the 2nd and the 3rd are DT, implemented with switched capacitor (SC) circuits. The third order structure fulfills the high resolution and linearity requirements with an oversampling ratio of R=512. A CT input integrator also helps to reduce the aliasing effects. A clock jitter of the return to zero (RZ) DAC in the feedback could limit the signal-to-noise ratio (SnR) [24] of the complete modulator, which is a well-known fact related to the CT ΣΔ ADCs; the reason is a big step of a feedback signal. The step can be reduced using a multi-bit DAC in the feedback, but the consequence would be a nonlinearity of the whole ADC, limited to approx. 10 bits because of matching accuracy of the CMOS process. This problem can be solved using FIR-DAC (finite-impulse-response digital to analog converter), which on one hand reduces the size of the steps of the internal DAC, and on the other hand keeps the linearity characteristics of a one bit DAC because it is composed of multiple one bit DACs with different weights. A simplified circuit diagram of the hybrid CT-DT modulator is presented in Figure 7. It is composed of a pair of CT integrators with a multi-bit feedback FIR-DAC with transfer function *F*(*z*), a common mode feedback circuit that keeps the outputs of the first integrators at selected common mode voltages and the input chopper with the multiplexer (which is part of the chopper structure). Two DT SC integrators, a 1-bit quantizer, and a digital clock form generator CFG follow it. The compensation filter IntEz with transfer function *K*(*z*) is connected to the input of the 2nd integrator. The FIR-DAC reduces the step size, but keeps the linearity of the DA converter unchanged, so the structure is less sensitive to the clock jitter. Unfortunately, the transfer function of the FIR-DAC changes the behavior of the modulator, so it must be compensated using the compensation filter *K*(*z*)*.* Figure 8 shows the simplified circuit diagram implementation of the FIR-DAC filter. The delayed bit-stream outputs (B0 through B15) control the switches of the DAC. The currents that flow into or out of the first integrator are: $I_{p}\left(n\right)=-I_{n}\left(n\right)=\sum_{k=0}^{k=15}B\left(n-k\right)\cdot I_{k}$, where $I_{k}$ are individual current weights and B(n−k) is the value of the bit-stream {±1} at instant k. The reference current is adjusted during automatic calibration procedure at power-up, so all currents and so the coefficients of the FIR-DAC filter with transfer function F(z) are adjusted. 

**Figure 9:**
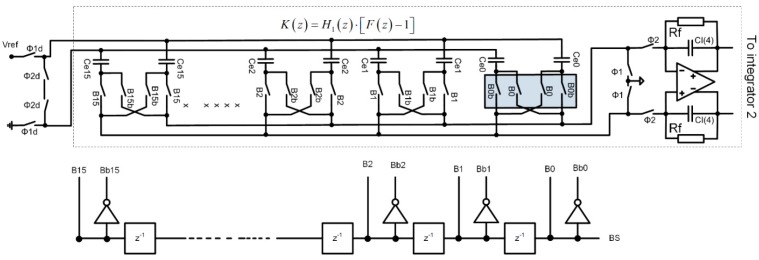
S-C implementation of compensation filter K(z) with a switched capacitor circuit.

Figure 9 shows SC implementation of the compensation filter *K*(*z*), which consists of the SC integrator shown on the right part of Figure 9, and the FIR filter implemented with SC circuits. In reality, the SC integrator also includes feedback resistors Rf, which have a negligible effect on the main transfer function, but keeps the operating point of the SC integrator stable.

### 4.2. Modelling and Simulations

A complete photo-current-to-digital converter is modeled before the circuit design on a high hierarchical level using Matlab/Simulink, where the most important non-idealities of the analog front end (AFE) like: finite gain, gain-bandwidth, slew-rate, kT/C, 1/f noise, and other thermal noise sources—offset, jitter, and component mismatches—are included [25,26]. At the same time, a high-level model of the corresponding DSP with finite word-length effects is used. 

**Figure 10:**
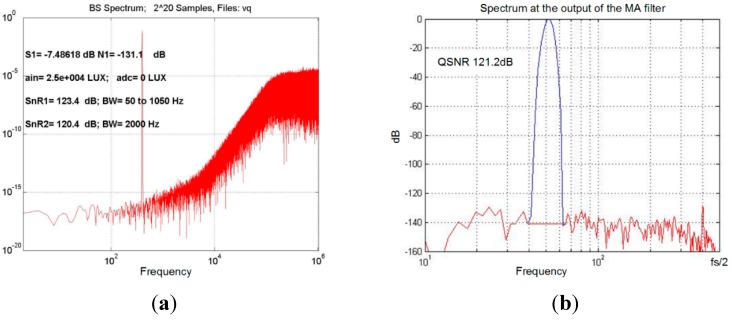
Spectra of the bit-stream for a sine-wave input light with an amplitude of 24 k Lux. The frequency is 1kHz and produces a 300 nA amplitude: (**a**) the spectrum of the bit-stream with chopping off; the most important non-ideal effects are included; (**b**) the spectrum at the moving average filter output with word-length reduced to 22 bits; the input signal is the same as for (**a**), but in this case LF chopping is on.

A simulated spectrum of a bit-stream at the output of the modulator, including all noise sources except jitter and including other non-ideal effects, is presented in Figure 10a for a 1 kHz sine-wave input light with intensity of 24 k Lux, producing a photo-current with 300 nA. In this simulation, the LF chopper is switched off. Figure 10b shows the spectrum at the output of the MA filter when LF choppers are switched on. The achieved signal-to-noise ratio SnR is over 120 dB in a 500 Hz bandwidth.

To prove the reduction of the jitter influence, the original modulator with 1 bit internal DAC and an improved version with a FIR-DAC and compensation filter with equal behavior and equal standard deviations of the clock jitter, were simulated under the same conditions. The first architecture produces signal-to-noise ratio (SnR) better than 110 dB for clock jitter, with a standard deviation that is smaller than $\sigma_{ji\_1}\leq 5\;\text{ps}$. The improved architecture with a multi-bit internal FIR-DAC reaches the same SnR at $\sigma_{ji\_2}\leq 49\;\text{ps}$, which is approx. 10-fold improvement. The jitter influence is reduced and the design of the phased-lock-loop (PLL) inside the chip is simplified. 

### 4.3. Circuit Design

**Figure 11:**
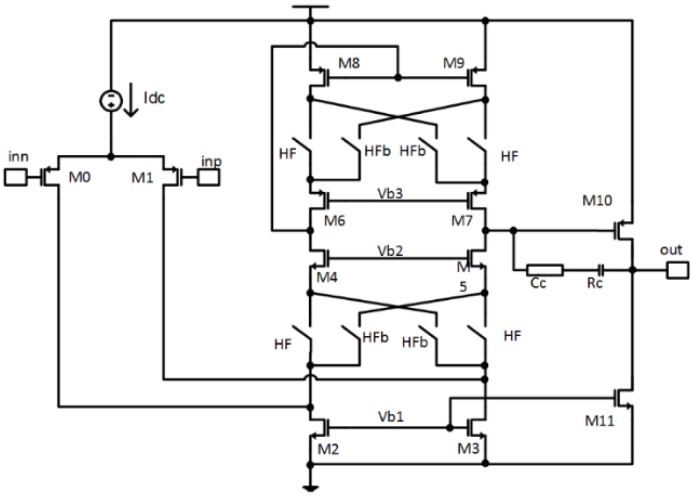
Simplified circuit diagram of the amplifier of the first CT integrator with HF chopper switches included.

Figure 11 shows a simplified circuit diagram of the opamp used in the first integrator with built-in HF chopper switches; it is single-ended to keep the virtual ground, and thus the cathode of each photo-diode at a fixed potential. The second and third SC integrators are built of folded cascode fully differential opamps without chopping and with less demanding characteristics. The demanding characteristics are required for the first opamp, since it must have high gain-bandwidth product, high open loop gain at $f_{hf\_chp}$ and low noise so that the SnR is not degraded.

The simulated characteristics of the first opamp are the following: $A_{0}\geq 100\;\text{dB}$, GBW≥20 MHz,$S_{R}\geq 10\text{ V}/\text{μs}$ and input referred noise density better than $V_{nd\_in}\leq 7\text{ nV}/\sqrt{\text{Hz}}$. It is possible to implement such an opamp at moderate power consumption because of the high frequency chopping of the folded cascode stage. The biggest power consumption of the whole modulator (approx. 70%) is needed in the first integrator because of the low noise requirements, high gain, and large gain-bandwidth product. 

### 4.4. System and Circuit Simulation Results

**Figure 12:**
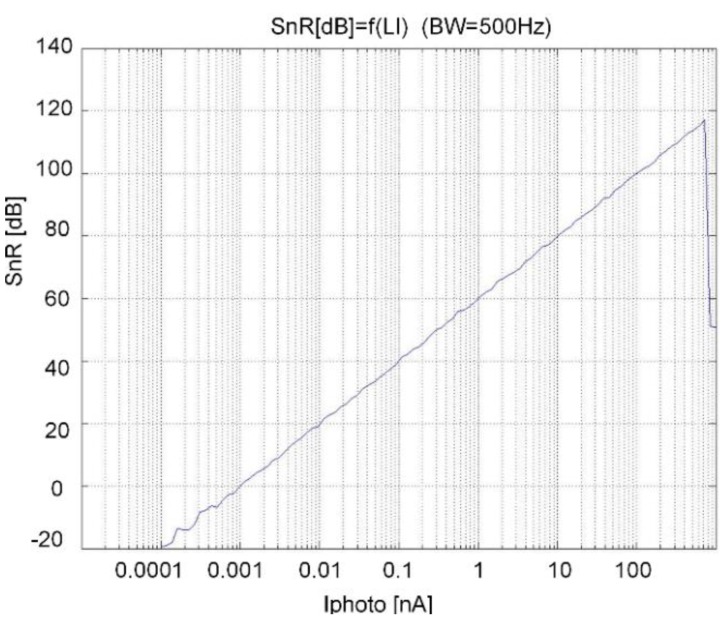
Circuit simulation result: SnR as a function of input light intensity with LF and HF choppers switched on.

Figure 12 shows the SnR at the output of the MA filter as a function of input photocurrent. The modulator (Figure 7), decimator, and MA filter are included in the simulation. The conditions are: LF and HF choppers are on, Reset is on, the input current is a sine-wave with 1 kHz frequency and with amplitudes in a range from 0.1 pA up to 1 uA. The photocurrent of 300 nA corresponds to a 24 k Lux light intensity. This simulation includes the most important non-ideal effects of the electronics and dark currents of the photodiodes, and show that the dynamic range is close to 120 dB, while the SnR at 300 nA photocurrent is approx. 110 dB in a 500 Hz bandwidth.

## 5. Color Signal Processing

**Figure 13:**
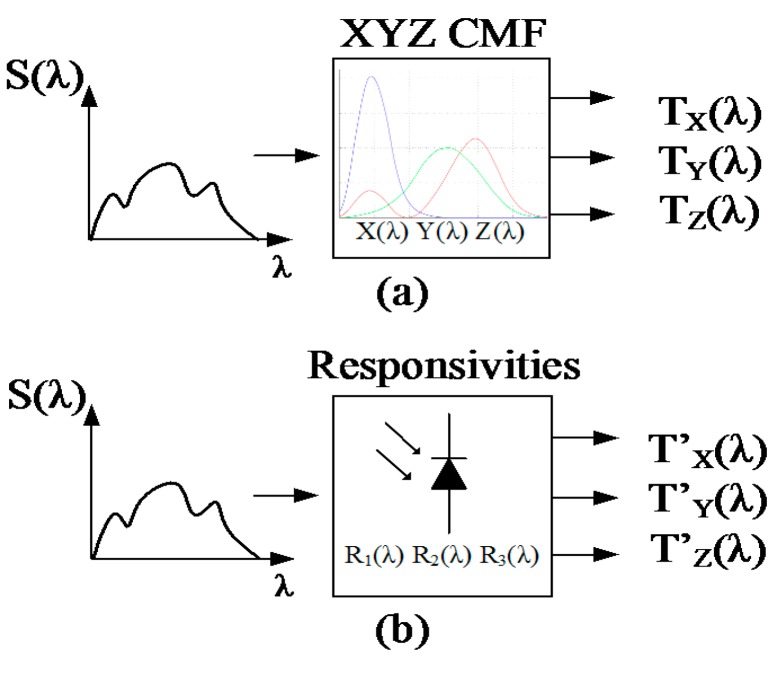
Generation of the T_X_, T_Y_, T_Z_ and T’_X_, T’_Y_, T’_Z_ stimuli.

In order to match the responsivity of the proposed color sensor to the commercial R,G,B or X,Y,Z color standard, a post processing of the photodiodes signal must be performed. The measured responsivity of the three-color photo sensors, as presented in Section 3.2.2 is used for the color transformations. When input stimulus S(λ) is applied to the X, Y, Z color matching function, a tristimulus output (T_X_, T_Y_, T_Z_) is generated, as shown in Figure 13. 

The tristimulus values T_X_, T_Y_, and T_Z_ can be expressed as integration over wavelength λ, where S(λ) is the received spectral power distribution of an illuminant. As S(λ) is usually measured at discrete intervals, the tristimulus values can be rewritten as the sum suggested in Equations (4)–(6):

(4) $$T_{X}=\int S\left(\lambda \right)x\left(\lambda \right)d\lambda \to T_{X}=\sum S\left(\lambda \right)x\left(\lambda \right)$$

(5) $$T_{Y}=\int S\left(\lambda \right)y\left(\lambda \right)d\lambda \to T_{Y}=\sum S\left(\lambda \right)y\left(\lambda \right)$$

(6) $$T_{Z}=\int S\left(\lambda \right)z\left(\lambda \right)d\lambda \to T_{Z}=\sum S\left(\lambda \right)z\left(\lambda \right)$$

The same input stimulus also generates three outputs (T’_X_, T’_Y_, T’_Z_) when applied to the responsivity of the three photodiodes of the color sensor, as shown in Figure 13b. T’_X_, T’_Y_, T’_Z_ can be obtained by the same method:

(7) $$T_{X,Y,Z}^{'}=\int S\left(\lambda \right)R_{1,2,3}\left(\lambda \right)d\lambda \to T_{X,Y,Z}^{'}=\sum S\left(\lambda \right)R_{1,2,3}\left(\lambda \right)$$

**Figure 14:**
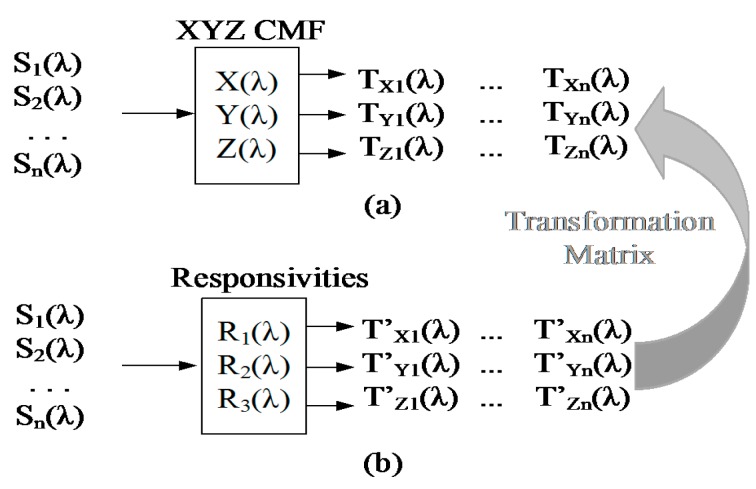
Use of training patterns to generate a transformation matrix suitable for different light spectra.

To allow the sensor to represent the light color of different spectra, a statistical training strategy can be used, as shown in Figure 14.

Several different input light sources, S_1_(λ) … S_n_(λ), are used as training stimuli to create a set of outputs with the X,Y,Z color matching function (Figure 14a) and a set of outputs defined by the color sensor responsivities (Figure 14b). A transformation matrix M with a minimum dimension of 3 × 3 can be used to transform T’_X_, T’_Y_, T’_Z_ into T_X_, T_Y_, T_Z_ according to Equation (8):

(8) $$\left[\begin{array}{ccc} T_{X1}^{'} & T_{Y1}^{'} & T_{Z1}^{'}\\ \vdots & \vdots & \vdots \\ T_{Xn}^{'} & T_{Yn}^{'} & T_{Zn}^{'} \end{array}\right]\cdot \left[\begin{array}{ccc} M_{11} & M_{12} & M_{13}\\ M_{21} & M_{22} & M_{23}\\ M_{31} & M_{32} & M_{33} \end{array}\right]=\left[\begin{array}{ccc} T_{X1} & T_{Y1} & T_{Z1}\\ \vdots & \vdots & \vdots \\ T_{Xn} & T_{Yn} & T_{Zn} \end{array}\right]$$

A matrix M with a minimized error can be calculated using the pseudo-inverse matrix ${\left(T_{X,Y,Z}^{'}\right)}^{-1}$. After training procedure, the obtained transformation matrix M can be used to transform the measured sensor output signals T’_X_, T’_Y_, T’_Z_ to the approximated X, Y, Z values T’_X,T_, T’_Y,T_, T’_Z,T_ using Equation (9). The transformed values T’_X,T_, T’_Y,T_, T’_Z,T_ have a relative error in relation to the ideal X, Y, Z tristimulus values to T_X_, T_Y_, T_Z_:

(9) $$\left[T_{X,T}^{'}T_{Y,T}^{'}T_{Z,T}^{'}\right]\cdot \left[\begin{array}{ccc} M_{11} & M_{12} & M_{13}\\ M_{21} & M_{22} & M_{23}\\ M_{31} & M_{32} & M_{33} \end{array}\right]=\left[T_{X}^{'}T_{Y}^{'}T_{Z}^{'}\right]$$

The relative error E is defined as (10):

(10) $$E=\frac{T_{X,Y,Z}-T_{XT,YT,ZT}^{'}}{T_{X,Y,Z}}\times 100\left[\%\right]$$

In order to minimize the relative error E, the dimensions of the transformation matrix M, the influence of the training stimuli, and the signals quantization were investigated. Light spectra of 15 different sources like halogen, fluorescent, LED, and sodium lamps were used for the training of the transformation matrix. The 15 light sources are further divided into six different training sets consisting of different combinations of the light sources. A training set including only the same type of light sources gives a low relative transformation error for the specified light source. For example, a training set of different LED light input spectra results in a relative error lower than 5% for LED lights, while the same transformation matrix can reach a relative error of 100% for a halogen or fluorescent input spectrum. The training set, including all 15 light sources, gives a quite high transformation error for an arbitrary input spectra. In order to reduce the error between transformed sensor values T’_X,T_, T’_Y,T_, T’_Z,T_ and the reference tristimulus values T_X_, T_Y_, T_Z_, a non-linear combination of the three sensor output values T’ like T’_X_ × T’_Y_, T’_Y_ × T’_Z_, or T’_X_ × T’_Z_ can be used to extend the sensor output information. The transformation matrix therefore needs to be extended to 6 × 3. However, since T_Y_, which is the luminance, is usually normalized to one, the transformation matrix M can be reduced to 4 × 3. The non-linear extension of the transformation matrix significantly decreases the relative error for the 15 light sources from 40% to below 10%. On the other hand, the increase of the transformation matrix also results in an increase of hardware for the mathematical operations from nine multipliers and six adders up to 12 multipliers and nine adders. 

Another key factor that affects the accuracy of the final output is the quantization of the input signals and coefficients of the transformation matrix. To keep the relative error low, the word-length of the transformation matrix should be higher than that of the input signal. Using the 4 × 3 transformation matrix, the relative error is less sensitive to the bit-width. Nevertheless, the error increases significantly if the word-length of the input signal is reduced to 6-bit and the transformation matrix to 10-bit. Therefore, a minimum of 14-bit input signal and 18-bit transformation matrix is necessary in order to ensure the acceptable influence of the quantization on the relative error. 

## 6. Experiments, Measurement Results, and Discussion

### 6.1. Color Sensor Measurements

**Figure 15:**
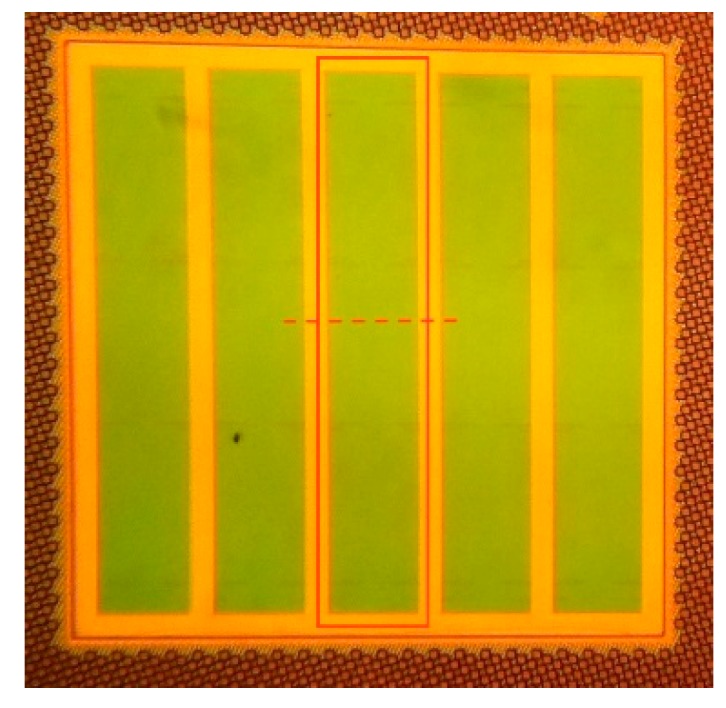
Photograph of the color-sensor test structure including indication of one sensor stripe and cut-line for cross-section.

A prototype of the presented color sensor structure was produced in a 130 nm standard CMOS technology. The structure consists of an array of five detectors stripes connected in parallel, with a total area of 250 µm × 250 µm, as shown in Figure 15. One detector stripe is marked with a rectangle and the cut-line for the cross-section shown in Figure 4 is indicated as dotted line. The photodiodes for dark current subtraction are equivalent to the detector shown in Figure 15, but fully covered by metal and placed next to the main sensor. The measurement setup includes a Newport Apex 70613NS illuminator and an Oriel Cornerstone 130 monochromator. A prototype of the presented color sensor structure was produced in a 130 nm standard CMOS technology. The structure consists of an array of five detectors stripes connected in parallel, with a total area of 250 µm × 250 µm, as shown in Figure 15. The photodiode currents are measured by Keithley source meters. Newport power meter Model 2936 is used to measure the optical power falling on the sensor as a reference in order to calculate the sensors responsivity. All the measurements were performed with the electrical contacts biased at 0 V DC voltage. 

**Figure 16:**
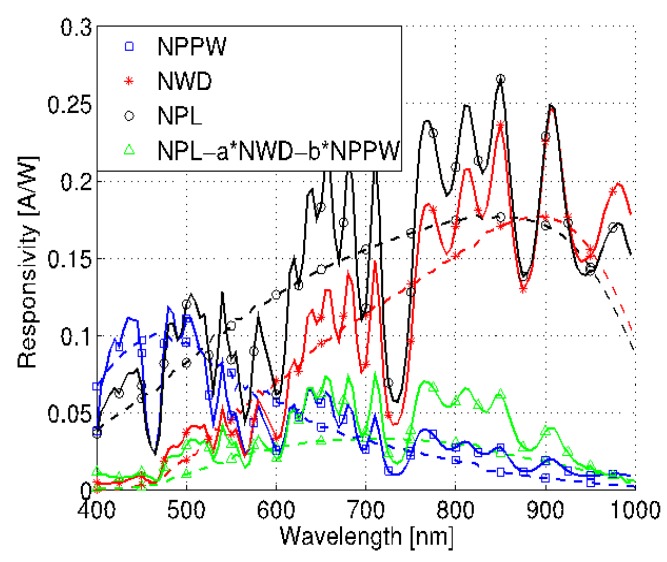
Measured responsivity of the three color sensor signals in comparison to simulation.

The measured spectral responsivities of the three photodiodes are presented in Figure 16, where the dashed lines represent the simulation results and the solid lines show the measurement. The measurements show significant spectral responsivity variations due to optical interference caused by the dielectric oxide stack. 

Even though the device responsivity can decrease for certain wavelengths due to dielectric interferences, there is a minor impact on the average detector responsivity behavior necessary for wide-band ambient light sensing. The average response shows good agreement between simulation and measurement. Measurements from several samples for different wide-band light sources show a minor variation of the sensors output signals, even though there is a significant variation of the spectral responsivity. Therefore in this case a dedicated responsivity characterization of each sensor is not necessary. In case of the measurement of the narrow-band light sources, like monochromatic light, the absolute value of the sensor output signals get more sensitive to technology variations, which might make a dedicated responsivity characterization necessary, increasing the overall sensor costs. In this case, a special processing of the optical windows - removing the oxide/nitride stack on top of the photo sensor - might pay off, because it reduces the optical interference and makes the sensor less sensitive to process variations. The pn-junction capacitances were measured for three different voltages, resulting in values between 3.9 pF and 28 pF, as shown in Table 1. The measured series resistance of the diodes is between 45 Ω and 60 Ω and the measured photodiode dark current is below 30 pA at room temperature.

**Table 1:** Measured pn-junction capacitance for different bias voltages.

Pn-Junction	Bias Voltage		
	2 V	1.5 V	0 V
P-substrate/N+ lighted	3.9 pF	4.2 pF	6 pF
P-substrate/N-well dark	5.5 pF	6 pF	8 pF
P-well/N+	18 pF	19.2 pF	28 pF

### 6.2. X,Y,Z Color Transformation

**Figure 17:**
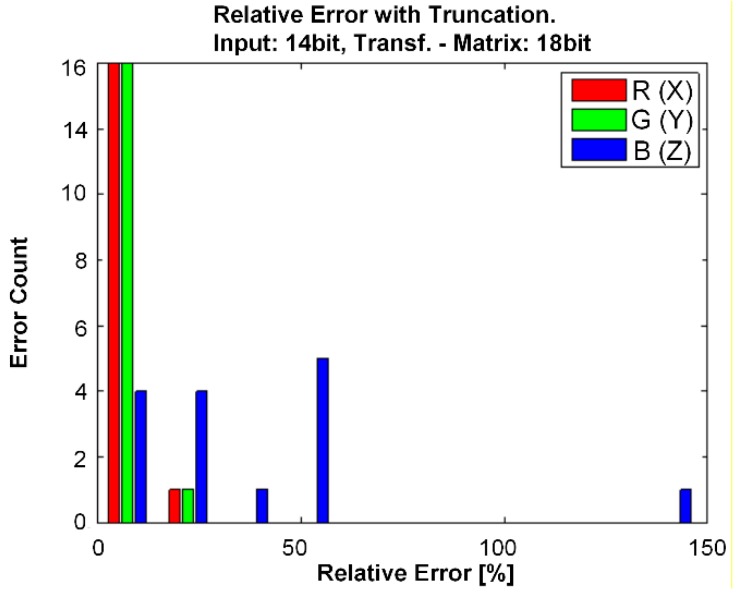
Relative error of X, Y, Z for 15 different light sources and 14-bit ADC.

Using the light training spectra of the 15 lamps, the sensor tristimulus T’_X,Y,Z_ is calculated based on measured sensor responsivities. Also, the ideal tristimulus response T_X, Y, Z_ based on the CIE CMFs are obtained and a 4 × 3 transformation matrix M is generated. After a quantization of the transformation matrix M and the sensor outputs T’_X,Y,Z_ to 18-bit and 14-bit, respectively, the transformation was performed to calculate the normalized sensor tristimulus values T’_XT,YT,ZT_. The relative error between T_X,Y,Z_ and T’_XT,YT,ZT_ is presented in the histograms of Figure 17. 

It can be observed that the error is smaller than 10% for 14 out of 15 “green” or Y-values (T’_YT_) and “red” or X-values (T’_XT_). The “blue” or Z-value (T’_ZT_) can achieve a very high relative high error, up to 150%. One reason for the high “blue” error is that the relative variation of the three sensor responsivities at low wavelengths is very low, which makes the pseudo-inverse matrix operation, and so the average error of the transformation matrix elements high. An improvement of the sensor responsivity at low wavelengths could, for example, be realized by stripe-shaped photodiodes as reported in [9]. For the presented sensor, a resolution of at least 14-bit of the three sensor signals is necessary. An improved sensor with more than three output signals can be realized [27], where the signal quantization has a significantly higher influence on the measurement error. Therefore, a high-resolution sensor data is mandatory, which explains why we built a 22-bit ADC.

### 6.3. Measurements of the Photocurrent-to-Digital Converter

The measurements of the photocurrent-to-digital converter were performed on the same test chip as the complete sensor measurements. One pair of multiplexer inputs are through the ASIC pads connected externally to the precision, low noise current source (Keithley model 6221 DC-AC current source). The measurement parameters were the same as for the simulations (see Figure 10b and Figure 12). 2^20^ samples of the BS and 2^8^ outputs of the MA filter were stored to the PC for each amplitude and the FFT analysis at different amplitudes were performed; the signal-to-noise ratio for each amplitude was calculated, and at the end the dynamic range was determined. The results of the measurements of the photo-current-to-digital converter are: $SnR\left(I_{in}=300\;\text{nA}\right)≅106\;\text{dB}$ and DR≅117 dB (compared to the results in Figure 12 the measurements are a bit worse).

### 6.4. Integrated Digital Color Sensor Measurements

**Figure 18:**
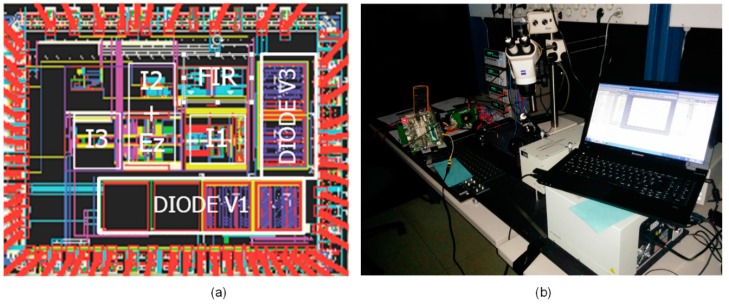
(**a**) Photo-micrograph of the chip including four photo-diodes input multiplexer and 3rd order modulator with FIR-DAC and compensation filter. DSP is implemented on the FPGA; (**b**) Optical measurement setup.

Figure 18a shows a photomicrograph of the integrated digital color sensor chip, which contains differently sensitive photo diodes (with area of 250 um × 750 um DIODE V3) connected to the input multiplexer and the integrated analog part of the incremental, 3rd order, photocurrent-to-digital ΣΔ ADC, where the important modules are marked (the area of the modulator is approx. 0.6 mm^2^). Figure 18b shows the optical measurement set-up; the chip in the package with integrated photo diodes and the modulator are located under the microscope, while FPGA with DSP can be observed on the left part of the picture together with other supporting instruments, for example power supplies, *etc*.

**Figure 19:**
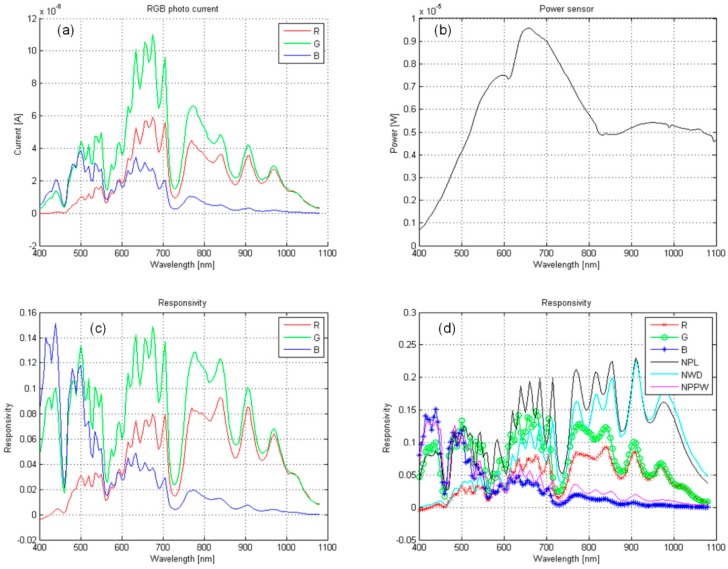
(**a**) Measured photocurrents of different photo-diodes; (**b**) Power of the light on the sensor as a function of the wavelength; (**c**) Responsivity of the complete detection system for three different photo-diodes; (**d**) A responsivity comparison between the integrated color detection system of this article and measurements obtained with the optical lab setup.

The measured results of the complete chip are presented in Figure 19.

(a) The photo currents of differently sensitive photo-diodes as a function of the wavelength of the incident light. This is the result of a measurement with the integrated detection system.
(b) Power of the incident light as a function of the wavelength (see Figure 19b).
(c) Responsivity calculated with $R=I_{photo}/P$ (P is the incident light power).
(d) The comparison of responsivity: integrated light detection system (lines with symbols) and the optical lab measurement setup, explained in Section 3.2.2.

A comparison of the measured results between the integrated light detection system and the optical lab measurement equipment show almost equal responses for (B, NPPW) diodes and significant differences in peaks between (R, NPL) and (G, NWD) diodes, however, the shapes are similar. This difference is attributed to the focus of the light source, which is an important factor in measurement accuracy. 

## 7. Conclusions

To conclude, an integrated CMOS color sensor based on lateral carrier diffusion is realized in 130 nm CMOS technology without any process modification, and together with a high resolution ADC and digital color signal processing is described in this article. The proposed sensor includes three photodiodes with different spectral responsivities, which is a prerequisite for a R,G,B or X,Y,Z color measurement. The photo-current-to-digital converter is integrated on the same silicon together with the sensors. A color signal processing based on a training strategy together with a least-mean-square pseudo-inverse matrix transformation is used to transform the outputs of the light-to-digital converter to approximated standardized CIE X,Y,Z tristimulus response. Currently, it is implemented on the field programmable gate array (FPGA) and can be easily integrated together with the other parts of the detection system.

As a further improvement, the lateral carrier diffusion effects of the proposed color sensor can be extended with additional field-plate structures, which enable a continuously tunable spectral responsivity [27]. This would enable more accurate colorimetric color measurement or even spectral reconstruction of input light, at the cost of higher signal processing effort and therefore chip area.
